# The Human NUP58 Nucleoporin Can Form Amyloids In Vitro and In Vivo

**DOI:** 10.3390/biomedicines9101451

**Published:** 2021-10-13

**Authors:** Lavrentii G. Danilov, Svetlana E. Moskalenko, Andrew G. Matveenko, Xenia V. Sukhanova, Mikhail V. Belousov, Galina A. Zhouravleva, Stanislav A. Bondarev

**Affiliations:** 1 Department of Genetics and Biotechnology, St. Petersburg State University, 199034 St. Petersburg, Russia; lavrentydanilov@gmail.com (L.G.D.); smoskalenko@mail.ru (S.E.M.); studentmag01@gmail.com (A.G.M.); sukhanovaxenia@gmail.com (X.V.S.); belousovmix@gmail.com (M.V.B.); 2 St. Petersburg Branch, Vavilov Institute of General Genetics, Russian Academy of Sciences, 199034 St. Petersburg, Russia; 3 Laboratory for Proteomics of Supra-Organismal Systems, All-Russia Research Institute for Agricultural Microbiology, 196608 St. Petersburg, Russia; 4 Laboratory of Amyloid Biology, St. Petersburg State University, 199034 St. Petersburg, Russia

**Keywords:** protein aggregation, human amyloids, Congo Red, evolution, nucleoporins, C-DAG, ArchCandy

## Abstract

Amyloids are fibrillar protein aggregates with a cross-β structure and unusual features, including high resistance to detergent or protease treatment. More than two hundred different proteins with amyloid or amyloid-like properties are already known. Several examples of nucleoporins (e.g., yeast Nup49, Nup100, Nup116, and human NUP153) are supposed to form amyloid fibrils. In this study, we demonstrated an ability of the human NUP58 nucleoporin to form amyloid aggregates in vivo and in vitro. Moreover, we found two forms of NUP58 aggregates: oligomers and polymers stabilized by disulfide bonds. Bioinformatic analysis revealed that all known orthologs of this protein are potential amyloids which possess several regions with conserved ability to aggregation. The biological role of nucleoporin amyloid formation is debatable. We suggest that it is a rather abnormal process, which is characteristic for many proteins implicated in phase separation.

## 1. Introduction

Amyloids are fibrillar protein aggregates with cross-β structure, which can also possess a number of other unusual features, including resistance to detergent and protease treatment and interaction with specific dyes (Congo Red and thioflavin T and S). Notably, Congo Red staining followed by demonstration of apple-green birefringence is considered as solid evidence that aggregates form cross-β structure (for review, see [1,2,3,4]). Approximately 120 different proteins with amyloid properties are already known, and more than a hundred other proteins are called amyloid-like [4]. There are several examples of nucleoporins (Nups) among this variety. Fragments of yeast Nup49, Nup100, Nup116, and human NUP153 are supposed to form amyloid fibrils [5,6,7,8]. These facts led to an assumption that other Nups can also form amyloids, and the ability to form aggregates may be common for such proteins.

The nucleoporin protein family comprises more than three thousand proteins, most of which are essential for the formation of the nuclear pore complex (NPC) [9]. These proteins can be divided into three groups: membrane, scaffold, and barrier nucleoporins. The barrier Nups usually contain phenylalanine-glycine repeat (FG repeat) regions suggested to play a key role in macromolecular transport through NPC [10]. It is noteworthy that all Nups forming hydrogels or aggregates contain FG repeats. Human NUP58 protein is one of the most abundant barrier nucleoporins in NPC (48 molecules per complex) [11], but its ability to aggregate has not been studied. In this article, we analyzed the conservation of the amyloidogenic properties of its orthologs and the ability of this protein to form amyloid aggregates in vitro and in vivo.

## 2. Materials and Methods

### 2.1. Bioinformatic Analysis

The set of the NUP58 protein orthologs was obtained from the EggNOG 5.0 orthologs database (http://eggnog5.embl.de/, accessed on 24 November 2020) [12]. The analysis included all orthologs of the NUP58 protein from the Chordata taxonomic group. ArchCandy was used to predict the amyloidogenic properties of proteins (threshold value 0.575) [13]. The IUPred program (with the option “long”) was used to predict unstructured regions [14,15]. The protein regions with the IUPred score more than 0.3 were considered as unstructured. A protein or its part was considered amyloidogenic if at least one β-arch (based on ArchCandy predictions) with the score above the threshold was located in an unstructured region.

To find regions with conservative amyloidogenic properties, we performed the multiple sequence alignment using the MUSCLE algorithm [16] with an additional refinement step in the UGENE software (http://ugene.net/, accessed on 30 January 2020) [17]. Then, the sequences were filtered to remove duplicates for the same species. The certain sequence was excluded from the analysis (i) if it was removed from the NCBI databases, (ii) if it is short and covers only the C-terminal part of the protein (without FG repeats), (iii) if it was significantly longer compared to other sequences. The full list of EggNOG IDs of analyzed sequences is presented in the Appendix A. After filtration, sequences were realigned with MUSCLE. The conservation of amyloid properties at a particular alignment position was evaluated as the fraction of sequences in which the corresponding amino acid is included in the amyloidogenic region. This analysis was performed in R [18] with the Biostring package [19]. The tidyverse package, including ggplot2, was used for data rearrangement and plotting [20].

### 2.2. Plasmid Construction

The new plasmid pVSGW-ccdB for the C-DAG system compatible with the Gateway cloning was obtained as follows. The *ccdB* cassette flanked with *att*R and restriction sites (Bsp120I and XbaI) was amplified using pDest-527 (Addgene plasmid #11518) as a template (primer sequences are presented in the Appendix B). The PCR-product and pVS72 vector [21] were digested with Bsp120I/NotI and XbaI to insert the cassette into the plasmid. The ligation mix was transformed into *ccdB*-resistant strain DB3.1 (Thermo Scientific, Waltham, MA, USA). Plasmids pDONR221-Sup35NM and pDONR221-Sup35M were obtained by the Gateway cloning. The PCR products of corresponding sequences flanked with *att*B sites were inserted into pDONR221 (Thermo Scientific, #12536017) by BP reaction (BP Clonase™ II Enzyme mix, Thermo Scientific). These entry clones were used for pVSGW-Sup35NM and pVSGW-Sup35M construction by the LR reaction (LR Clonase™ II Enzyme mix, Thermo Scientific).

Plasmids pDEST527-NUP58 and pVSGW-NUP58 bearing the full-length NUP58 for protein purification and experiments in C-DAG system, respectively, were obtained by the LR reaction. Plasmids pDest-527 (Addgene plasmid #11518) and pVSGW-ccdB were used as destination vectors and pDONR221-NUP58 (Thermo Scientific Ultimate ORF Clone IOH4601) as the entry clone. To construct pVSGW-NUP58-1-213 corresponding protein coding sequence was PCR amplified with primers containing *att*B sites (primers sequences are listed in the Appendix B). The pDONR221-NUP58 plasmid was used as a template. The plasmid for C-DAG assay was then obtained using Gateway cloning, analogously to other plasmids.

### 2.3. Microbiological Procedures

Standard microbiological approaches and media were used for all manipulations with bacteria [22]. *Escherichia coli* strains TOP10 (Invitrogen) and DH5α [23] were used for cloning; the DB3.1 strain (Thermo Scientific) was used as a host for plasmids with *ccdB* cassette.

### 2.4. Protein Electrophoresis and Hybridization

The SDD-AGE was performed according to the published protocol with minor modifications [24,25]. Gels with 1.5–2% (*w*/*v*) agarose were run at 30 V for 200–240 min in all experiments. The SDS-PAGE was performed according to the previously published protocols [22]. SDS-PAGE with gel boiling [26] was performed to detect NUP58 in the aggregated and soluble fractions. β-mercaptoethanol (BME) was added to the final concentration 2% (*v*/*v*) in most experiments. Commercial monoclonal anti-His antibody was used to detect recombinant NUP58 (GE Healthcare Life Sciences, Pittsburgh, PA, USA, 27-4710-01). Coomassie gel staining was performed as described previously [22]. Briefly, the gel was boiled in the dye solution (0.25% (*w*/*v*) Coomassie G-250, 10% (*v*/*v*) acetic acid, 50% (*v*/*v*) ethanol) for 1 min. The excess of the dye was removed by subsequent boiling in water.

### 2.5. C-DAG System

For the experiments in the C-DAG system, the *E. coli* strain VS39 [21] was transformed with target plasmids, and transformants were selected on LB medium supplemented with ampicillin and chloramphenicol at 37 °C [22]. The overnight bacterial culture was diluted 100-fold and grown for one hour. The obtained cultures were plated on series of media: CR inducing plate (0.2% (*w*/*v*) L-arabinose, 0.1 mM IPTG, 10 μg/mL Congo Red dye, 200 μg/mL ampicillin and 25 μg/mL chloramphenicol), and the control plate contained only antibiotics. Plates were incubated at 26 °C for 5 days. Plasmids pVSGW-Sup35NM, pVSGW-Sup35M were used as a positive and negative control, respectively. Samples for polarization microscopy were prepared as follows. 20 μL of the cell’s suspension were applied on a slide and dried. The cells were covered with 5 μL of 50% (*v*/*v*) glycerol and analyzed with an inverted Leica DMI6000 microscope.

### 2.6. NUP58 Purification from E. coli and Fibril Preparation

*E. coli* strain BL21(DE3) [27] transformed with pDEST527-NUP58 was used for the protein purification. Overproduction of the recombinant protein was carried out in LB media with 100 μg/mL ampicillin and 1 mM IPTG. Cultures were grown at 37 °C for 6 h. NUP58 was purified in denaturing conditions in the presence of 8 M urea according to the protocols proposed for Sup35NM [28]. The purification was performed using only Ni-NTA agarose (Invitrogen, Thermo Fisher Scientific, Waltham, MA, USA, R901-15). The obtained NUP58 protein was diluted at least 100-fold into fibril assembly buffer (5 mM potassium phosphate pH 5.8, 150 mM NaCl) to a final protein concentration of 1 mg/mL. At these conditions, NUP58 spontaneously forms aggregates. Samples were incubated at 37 °C with slow overhead rotation (rotator Bio RS-24, Biosan, Riga, Latvia). To monitor amyloid fibril formation, aliquots were removed every 12 h up to 24 h of incubation. The rate of aggregated protein was estimated by SDS-PAGE with boiled and unboiled samples. Sup35NM aggregates were obtained as described previously [25].

### 2.7. In Vitro Congo Red Staining

The obtained NUP58 fibrils were analyzed for the Congo Red dye binding as follows. Samples were prepared by applying 20 µL of the NUP58 fibril solution with a concentration of approximately 2.5 mg/mL (the aggregates were pelleted and then resuspended in smaller volume) on glasses and drying it. Then, the Congo Red dye (dia-m, D573580) water solution at a concentration of 1 mg/mL was added to the sample and incubated for 10–15 min. The excess of the dye was washed out with 70% (*v*/*v*) ethanol. The sample was covered with 5 μL 50% (*v*/*v*) glycerol and analyzed with an inverted microscope Leica DMI6000. Sup35NM fibrils (5 mg/mL) and BSA (10 mg/mL, Promega, Madison, WI, USA, R396D) were used as positive and negative controls, respectively.

### 2.8. Transmission Electron Microscopy (TEM)

The negative staining with a 1% (*w*/*v*) aqueous solution of uranyl acetate was used for TEM. Samples were prepared by applying 5 µL of the NUP58 fibril solution with a concentration of 1 mg/mL or bacterial cell suspension from a CR inducing plate on a formvar coated grid, followed by washing with distilled water and drying. The samples were stained with the dye for 30 s. The excess of the uranyl acetate was removed with incubation in distilled water for 30 s. Jeol JEM-2100 (in vitro samples) or Jeol JEM-1400 (bacterial samples) transmission electron microscopes were used for the subsequent analysis.

### 2.9. Proteinase K Resistance Assay

Proteinase K (Helicon, Moscow, Russian Federation, Am-O706) was added to the monomeric and aggregated protein at a concentration of 0.5 µg/mL. The solutions were incubated for 60 min at 26 °C. The reaction was terminated by the addition of PMSF to the final concentration of 8.3 mM. The amount of undigested protein was analyzed with SDS-PAGE.

### 2.10. Thioflavin T Staining

Thioflavin T (Sigma-Aldrich, T3516) was added to the protein samples to a final concentration of 12 μM. Fluorescence measurements were performed on a CLARIOstar Plus (BMG Labtech, Ortenberg, Germany) spectrofluorometer in microplates. Fluorescence emission spectra were recorded from 478 to 600 nm wavelengths (slit width 10 nm, gain 750) with 450 nm excitation wavelengths (slit width 16 nm). BME was added to a final concentration of 2% (*v*/*v*) where indicated.

## 3. Results and Discussion

### 3.1. NUP58 and Its Orthologs Are Potential Amyloids

**Figure 1:**
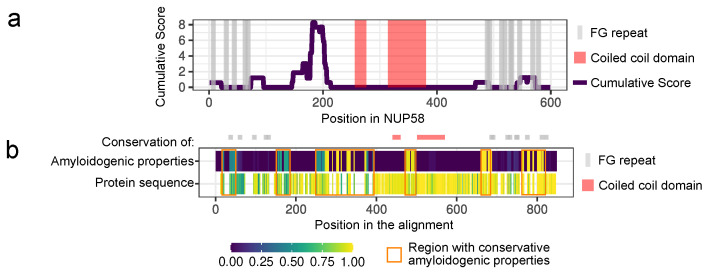
Human NUP58 and its orthologs are potential amyloids: (**a**) Amyloidogenic regions in NUP58. The ArchCandy Cumulative Score reflects the ability of the protein to form β-arches. (**b**) The heatmap demonstrates the sequence conservation of the NUP58 orthologs (spaces designate columns of the alignment, containing a lot of gaps) and the fraction of the amyloidogenic sequences in a certain position of the alignment. Orange rectangles mark regions with conservative amyloidogenic properties. FG repeats and coiled coil domains are marked with gray lines and red rectangles, respectively; their positions correspond to the human NUP58 protein.

We used the ArchCandy program [13] to analyze the amyloidogenic properties of NUP58 and its orthologs. The unique feature of this tool is the ability to predict special structural motifs, called β-arches, which are found in almost all amyloids [13,29]. Another hallmark of ArchCandy is its high accuracy [13,30,31]. Unstructured protein regions have a higher tendency to aggregate than folded domains. To take this into account, we used the IUPred tool [14,15] to predict unstructured regions in the analyzed proteins. We considered as amyloidogenic those fragments of the proteins that were unstructured and could form at least one β-arch. This analysis revealed five amyloidogenic regions in human NUP58 and demonstrated that this protein is a potential amyloid. The most amyloidogenic fragment is located between 146 and 213 amino acids and does not overlap with the coiled coil domains of the protein (Figure 1a). These domains are required for protein interactions in the NPC subcomplexes (with NUP62 and NUP54) which form a selective barrier in the pore [32].

Then, we repeated the analysis for the known orthologs of NUP58 across Chordata (total 101 proteins, corresponding EggNOG IDs are listed in the Appendix A) and found that all of them are also amyloidogenic. Further, we compared the localization of the predicted amyloidogenic regions in different proteins based on the multiple alignment. These results allowed us to find several fragments that are presented in almost all sequences and are aggregation-prone (Figure 1b). Remarkably, the conservation of amyloidogenic properties in these regions is even higher than the similarity of protein sequences. The latter can reflect the significance of the tendency to aggregate in evolution.

### 3.2. NUP58 Protein Demonstrates Amyloid Properties In Vitro

**Figure 2:**
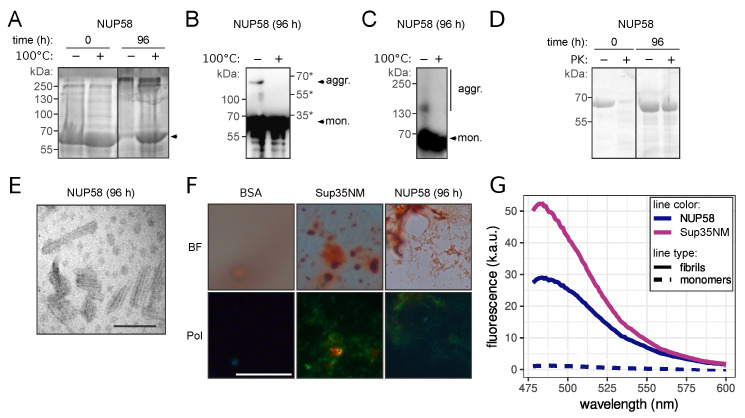
Human NUP58 protein forms amyloid aggregates in vitro: (**A**–**C**) Visualization of NUP58 detergent-resistant aggregates with different approaches: SDS-PAGE (**A**), SDS-PAGE with additional boiling (**B**), and SDD-AGE (**C**). NUP58 samples were compared at the starting point (0 h) and after four days (96 h) of incubation in non-denaturing conditions. Arrowheads mark the position(s) of NUP58. The plus and minus symbols correspond to the boiled and unboiled samples in all panels. Numbers mark the positions of the protein molecular weight markers (kDa), the star (*) marks the protein ladder added after the gel boiling. Coomassie staining (**A**) or Western blotting with monoclonal anti-His antibodies (**B**,**C**) were used for the protein visualization. (**D**) The treatment of monomeric and aggregated NUP58 protein with proteinase K (PK). Presence of the protein was detected by gel staining with Coomassie. (**E**) Electron microphotograph of the NUP58 fibrils. The scale bar equals 100 nm. (**F**) NUP58 fibrils staining containing Congo Red. The microphotographs were obtained under a transmitted (BF) and cross-polarized (Pol) light. Sup35NM fibrils and BSA solution were used as positive and negative controls, respectively. The scale bar equals 20 µm. (**G**) Fluorescent spectra of protein samples stained with ThT. Median values from three replicates are present.

The key property of amyloids is the cross-β structure of aggregates. Such structure can be demonstrated with NMR and electron or X-ray diffraction experiments. However, the staining with Congo Red and demonstration of apple-green birefringence in the polarized light is considered as an appropriate analogue. Besides, many amyloids possess other special features such as detergent and protease resistance (for review, see [1,2,3,4]). To investigate the amyloid properties of NUP58 protein, we have purified the full-length protein fused with His_6_ tag from *E. coli* cells. Amyloid aggregates are high molecular weight complexes resistant to the incubation with SDS at room temperature, and they cannot enter polyacrylamide gel. Thus, their presence can be detected by the difference in the protein amount between boiled and unboiled samples detected in the gel (boiling in the SDS containing buffer completely dissolves the aggregates to monomers). With this approach, we found that after four days of incubation (see Materials and Methods for details), NUP58 had formed SDS-resistant aggregates (Figure 2A). The presence of NUP58 in the aggregate fraction was also demonstrated using SDS-PAGE with additional boiling (Figure 2B), and the small NUP58 aggregates and oligomers were detected using SDD-AGE (Figure 2C) (see Materials and Methods for details). Further, we investigated the resistance of aggregated and monomeric NUP58 to the protease treatment. The addition of Proteinase K (PK) into the solution of NUP58 aggregates had a negligible effect on the protein amount after 30 min of incubation. At the same time, the monomeric protein was almost completely digested at the same conditions (Figure 2D).

**Figure 3:**
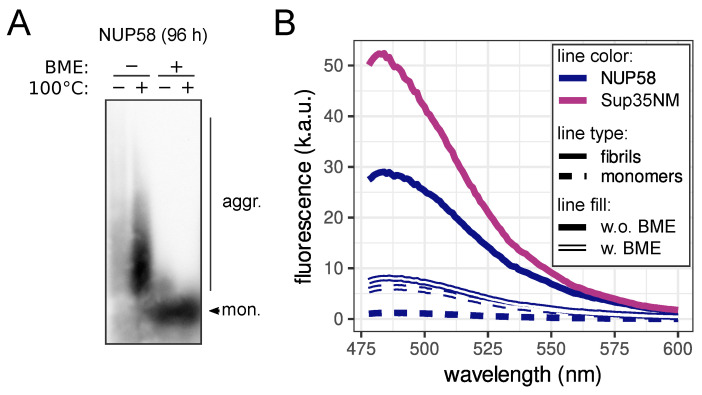
Large aggregates of NUP58 protein are stabilized by disulfide bonds: SDD-AGE (**A**), and fluorescent spectra of ThT (**B**) in protein samples with or without BME (spectra of samples without BME from Figure 2 are presented for comparison). The plus symbols mark samples with BME or boiled ones. Western blotting with monoclonal anti-His antibodies was used for the protein visualization after SDD-AGE. Median values from three replicates are present on the plot.

Further analysis of these samples with transmission electron microscopy (TEM) revealed that aggregates had a fibrillar morphology (Figure 2E), which is common for amyloids. The remarkable feature of investigated complexes was their small size. The length of the fibrils was approximately 100 nm, which was consistent with the estimated weight of aggregates on SDD-AGE (approximately several hundreds of kilodaltons). Staining of the NUP58 fibrils with Congo Red led to the characteristic apple-green birefringence in the polarized light, similar to well-known amyloid aggregates of Sup35NM [33] (Figure 2F). The presence of NUP58 aggregates significantly increased the fluorescence of the thioflavin T (ThT) probe (Figure 2G). Thus, we conclude that human NUP58 forms amyloid aggregates, which are predominantly presented as oligomers. We might speculate that these small aggregates include several protein molecules that are somehow linked to the number of NUP58 molecules in the NPC [11]. Detailed analysis of the NUP58 solution revealed two forms of aggregates, which are different in size, resistance to BME, and ThT staining. We tested the resistance of NUP58 fibrils to “cold” SDS in the absence of BME in the loading buffer for SDD-AGE (Figure 3A). Surprisingly, we detected large aggregates, which are resistant to the boiling in SDS; however, we observed the accumulation of aggregates with smaller size after heating. As BME reduces disulfide bonds, we concluded that these aggregates are rather polymers, stabilized by disulfide bonds, which explains their stability. An analogous situation was previously described for the β2-microglobulin protein, whose amyloid aggregates are also stabilized by disulfide bonds [34]. Then, we investigated ThT staining of NUP58 aggregates in the presence of BME. The addition of the reducing agent significantly decreased the dye fluorescence; however, the difference between aggregated and monomeric proteins was detectable (Figure 3B).

### 3.3. Human NUP58 Protein Demonstrates Amyloid Properties in C-DAG System

**Figure 4:**
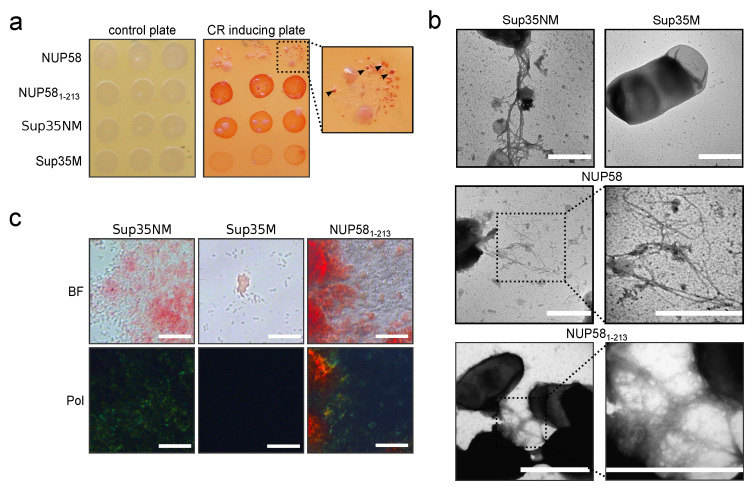
The full-length NUP58 and its fragment from 1 to 213 a.a. demonstrate amyloid properties in the C-DAG system: (**a**) Bacterial cells, overproduced NUP58 or NUP58_1-213_ are red on the medium containing Congo Red (CR inducing plate). The control plate, containing only antibiotics, was used to check the amount of plated cells. For the full-length NUP58 protein, we could detect small red colonies, marked with arrows. (**b**) TEM microphotographs of cells with fibrils formed by NUP58 protein and its fragment. Scale bar equals 1 μm. (**c**) Bacterial cells that produce NUP58_1-213_ protein demonstrated an apple-green birefringence in polarized light. The microphotographs were obtained under a transmitted (BF) and cross-polarized (Pol) light. The scale bar equals 50 μm. Plasmids encoding Sup35NM and Sup35M were used as positive and negative controls, respectively.

Previously, it was hypothesized that under correctly chosen conditions it is possible to obtain an amyloid form of absolutely any protein in vitro [35]. Due to this, we performed additional experiments in vivo to investigate the ability of NUP58 to form amyloid aggregates. An approach called C-DAG (curli-dependent amyloid generator) was proposed to analyze amyloid properties of proteins in bacterial cells [21]. The formation of amyloid aggregates is detected by (i) the red color of cells grown on the media containing Congo Red, (ii) their apple-green birefringence in cross-polarized light, and (iii) the appearance of fibrillar aggregates on the cell surface. The amyloidogenic (NM) and non-amyloidogenic (M) regions of Sup35 were used as positive and negative controls in this system [21]. In this work, we modified an original plasmid by adding a *ccdB* cassette flanked by *att*P sites for Gateway cloning. Control constructs, based on the new plasmid, were compared to published vectors [21] in all of the above-mentioned tests, and only minor differences were found (data not shown). The overproduction of the full-length NUP58 decreased the growth of bacterial cells. Previously, it was described by the authors of the assay that large proteins are less likely to be produced in the C-DAG system. However, we observed small red colonies on the media containing Congo Red (Figure 4a). Cell material, collected from these colonies, was sufficient to prepare samples for TEM, but not for polarized microscopy. The TEM demonstrated the presence of fibrillar aggregates on the surface of the cells, overproducing human NUP58 (Figure 4c). These results provided the first evidence that the investigated protein can aggregate in vivo.

The decreased growth of the bacteria with the overproduced protein yields a small number of cells, which was insufficient for the analysis using polarization microscopy. To solve this problem, we obtained the plasmid with the truncated protein bearing the main amyloidogenic region (Figure 1a). The overproduction of NUP58_1-213_ had no effect on bacterial growth, and the cells were red on the medium containing Congo Red (Figure 4a). When analyzing these cells using a polarizing microscope, we could observe apple-green birefringence (Figure 4c). Finally, using TEM, we showed that a fragment of NUP58_1-213_ also forms fibrils (Figure 4b).

Our results for the first time demonstrate that the human NUP58 protein can form amyloids in vitro and in vivo. NUP58 aggregates possess most of the characteristic properties. They are fibrillar and resistant to the detergent and protease treatment. Moreover, these fibrils were stained with amyloid-specific dyes (ThT and Congo Red) and demonstrated characteristic properties: an increase in fluorescence and apple-green birefringence, respectively (Figure 2). Interestingly, we detected two forms of NUP58 aggregates in the solution with different sizes (Figure 3a). The large aggregates were sensitive to the treatment with BME, which reduces disulfide bonds and can thus be called polymers. We suppose that the two cysteine residues in NUP58 at positions 252 and 521, located outside of the predicted amyloidogenic region (1-213 aa, Figure 1a), are responsible for this. The relationship between polymers and aggregates of NUP58 is unclear and needs to be investigated. It is possible that polymers are composed of small oligomers, linked by disulfide bonds. However, we are also unable to exclude that polymers and oligomers are independent of each other and were both present in the solution.

NUP58 also demonstrates amyloidogenic properties in vivo at least in the bacterial C-DAG system. Cells producing full-length NUP58 or its amyloidogenic part NUP58_1-213_ can form amyloid aggregates on the surface. This was confirmed by red colony color on the media containing Condo Red, apple-green birefringence of cells in polarized light, and protein fibrils on the surface, revealed with TEM (Figure 4). Since both cysteines are located outside of the NUP58 fragment 1-213 aa, which forms amyloids in the C-DAG system, we conclude that these residues are not essential for the amyloidogenesis of the protein. Therefore, we suggest that small oligomers detected in vitro in the presence of BME were also amyloid.

Nups with FG repeat regions form a selective barrier inside NPC, filling the central channel by their unstructured domains. Small molecules easily pass this filter, but large cargoes require interaction with specific nuclear transport receptors, which can pass through the barrier due to their ability to interact with the FG repeats [36]. Previously, it was considered that FG repeat regions of yeast Nsp1 and other Nups form stable hydrogel inside NPC [7,11,37,38]. Further investigations demonstrated the existence of intermolecular β-sheets, a specific feature of amyloids, in the hydrogels [11]. Amyloid fibrils were proposed to play an important role in the formation of the hydrogels by yeast Nup49 and human NUP153 FG repeat regions [6]. Moreover, predicted prion domains of yeast Nsp1, Nup42, Nup49, Nup57, Nup100, and Nup116 formed aggregates in vivo. In almost all cases, they were resistant to SDS (Nup57 was an exception), and only aggregates of Nup100 and Nup116 fragments were stained with ThT in vitro [5]. Thus, a number of works demonstrated the formation of stable hydrogels or amyloid fibrils by Nups and discussed the role of these strucutres in the formation of the selective barrier. The recent study [39] presented an opposite point of view. A liquid–liquid phase separation, but not hydrogel formation, was shown to be essential for the formation of the selective barrier. The phase separation of Nups with FG repeats and their interaction with cargoes were investigated on a millisecond time scale that allowed to monitor the formation of dynamic droplets by Nup49 [39]. From this point of view, the amyloid aggregation of Nups is an abnormal process. Analogous examples of the dualistic role of protein phase separation are known. Many RNA-binding proteins are able to form liquid droplets, which are essential for the maintenance of membrane-less organelles, but also amyloids, implicated in the development of pathological processes (for review, see [4]). Thus, the presence of amyloids inside NPC and their functional role in nuclear transport is debatable and, we suppose, unlikely.

Our bioinformatic analysis revealed that all orthologs of the NUP58 protein are also potential amyloids. We have also found a fragment which is amyloidogenic in many orthologs and located outside of the FG repeat region, which is supposed to play a key role in formation of selective barrier [40,41,42]. Notably, the conservation of the protein sequence in this fragment is lower than the conservation of amyloidogenic properties (Figure 1b). This fact may indicate that the formation of amyloids inside NPC is beneficial. The same idea was previously proposed based on evidence of amyloid formation by human and yeast nucleoporins [6]. However, it is more likely that the same sequences are required for phase separation and amyloid formation. Thus, we suggest that the identified fragment of NUP58 with a conservative tendency to aggregation may play an important role in the formation of selective barrier in NPC.

## Data Availability

Analyzed protein sequences are publicly available in the EggNOG database (http://eggnog5.embl.de/, accessed on 24 November 2020).

## Sample Availability

Plasmids are available from the authors.

## Abbreviations

The following abbreviations are used in this manuscript:

C-DAG	curli-dependent amyloid generator
FG repeat	phenylalanine-glycine repeat
NPC	nuclear pore complex
Nups	nucleoporins
PK	proteinase K
BME	β-mercaptoethanol
ThT	thioflavin T
SDS	sodium dodecyl sulfate
TEM	transmission electron microscopy

## Appendix A

List of EGGNog ID of NUP58 sequences:

10029.XP_007609480.1, 10036.XP_005075386.1, 10042.XP_006986393.1, 10090.ENSMUSP 00000038716, 10116.ENSRNOP00000017204, 10141.ENSCPOP00000011058, 10160.XP_004631 170.1, 10181.XP_004854885.1, 1026970.XP_008823213.1, 106582.XP_004546565.1, 109478.XP_0 05865972.1, 118797.XP_007471528.1, 128390.XP_009475149.1, 132908.ENSPVAP00000010480, 13616.ENSMODP00000010712, 13735.ENSPSIP00000010156, 144197.XP_008274508.1, 176946. XP_007439043.1, 181119.XP_005516327.1, 185453.XP_006832113.1, 215358.XP_010746949.1, 225400.XP_006753751.1, 244447.XP_008305689.1, 246437.XP_006159593.1, 27679.XP_0039197 89.1, 28377.ENSACAP00000002255, 28737.XP_006894193.1, 29073.XP_008690376.1, 29078.X P_008151582.1, 303518.XP_005722522.1, 30611.ENSOGAP00000003400, 31033.ENSTRUP000 00003387, 32507.XP_006794762.1, 34839.XP_005406388.1, 42254.XP_004604549.1, 43179.ENSSTOP00000003150, 482537.XP_008570061.1, 48698.ENSPFOP00000009818, 51337.XP_00467 0589.1, 51511.ENSCSAVP00000015277, 59463.ENSMLUP00000011639, 59538.XP_005975544.1, 59894.ENSFALP00000009059, 60711.ENSCSAP00000014019, 61622.XP_010358479.1, 61853.E NSNLEP00000016968, 69293.ENSGACP00000003141, 72004.XP_005888809.1, 7719.XP_00212 5735.1, 7897.ENSLACP00000018156, 7918.ENSLOCP00000004824, 7955.ENSDARP000001057 27, 79684.XP_005355465.1, 7994.ENSAMXP00000008000, 8010.XP_010894764.1, 8049.ENSGMOP00000002410, 8081.XP_008394338.1, 8083.ENSXMAP00000000749, 8090.ENSORLP00000 008650, 8128.ENSONIP00000018244, 8153.XP_005912082.1, 8469.XP_007059198.1, 8479.XP_00 5286751.1, 8496.XP_006274483.1, 885580.XP_010622097.1, 89462.XP_006071015.1, 9031.ENSGALP00000027595, 9258.ENSOANP00000014398, 9305.ENSSHAP00000015928, 9315.ENSMEUP00000003636, 9361.ENSDNOP00000004101, 9361.ENSDNOP00000011237, 9365.XP_007 528674.1, 9371.XP_004714876.1, 9402.XP_006915895.1, 9478.XP_008056345.1, 9483.ENSCJAP00000035743, 9541.XP_005585566.1, 9544.ENSMMUP00000024226, 9555.ENSPANP00000 002975, 9593.ENSGGOP00000003617, 9597.XP_003818400.1, 9598.ENSPTRP00000009744, 9601.ENSPPYP00000005949, 9606.ENSP00000371155, 9612.ENSCAFP00000010506, 9646.ENSAMEP00000015018, 9685.ENSFCAP00000001467, 9694.XP_007074467.1, 9707.XP_00440956 2.1, 9713.XP_006738183.1, 9733.XP_004281831.1, 9739.XP_004319890.1, 9767.XP_007164343.1, 9778.XP_004386620.1, 9785.ENSLAFP00000009874, 9818.XP_007940726.1, 9913.ENSBTAP0000 0032816, 9940.ENSOARP00000014067, 9978.XP_004577762.1, 9986.ENSOCUP00000025107.

## Appendix B

**Table A1:** Primers used in work: Capital letters indicate *att*B recombination or restriction sites.

Primers Name	Sequence	Application
NUPL1-F-1-213	GGGGACAAGTTTGTACAAAAAAGCAGGCTccatgtccacaggg	Amplification of the *NUP58* fragment, corresponding to 1–213 aa, with *att*B sites.
NUPL1-R-1-213	GGGGACCACTTTGTACAAGAAAGCTGGGTctaattgcctgcagttg	Amplification of the *NUP58* fragment, corresponding to 1–213 aa, with *att*B sites.
attB1-SUP35-F	GGGGACAAGTTTGTACAAAAAAGCAGGCTcaacaatgtcggattcaaacc	Amplification of the *SUP35* fragment, corresponding to Sup35NM, with *att*B sites.
attB2-SUP35M-stop-R	GGGGACCACTTTGTACAAGAAAGCTGGGTtcaatcgttaacaacttcgtcatcca	Amplification of the *SUP35* fragments, corresponding to Sup35NM and Sup35M, with *att*B sites.
attB1-SUP35M-F	GGGGACAAGTTTGTACAAAAAAGCAGGCTcaatgtctttgaacgactttcaaaag	Amplification of the *SUP35* fragment, corresponding to Sup35M, with *att*B sites.
Bsp120I-ccdB-F	tataGGGCCCCAcaccatcaccatcaccatagatctg	Amplification of the *ccdB* cassette.
XbaI-ccdB-R	tataTCTAGAgctatctagctagcgatatcaccac	Amplification of the *ccdB* cassette.

## References

[1] Benson M.D., Buxbaum J.N., Eisenberg D.S., Merlini G., Saraiva M.J.M., Sekijima Y., Sipe J.D., Westermark P. (2020). Amyloid nomenclature 2020: Update and recommendations by the International Society of Amyloidosis (ISA) nomenclature committee. Amyloid.

[2] Sergeeva A.V., Galkin A.P. (2020). Functional amyloids of eukaryotes: Criteria, classification, and biological significance. Curr. Genet..

[3] Rubel M.S., Fedotov S.A., Grizel A.V., Sopova J.V., Malikova O.A., Chernoff Y.O., Rubel A.A. (2020). Functional mammalian amyloids and amyloid-like proteins. Life.

[4] Matiiv A.B., Trubitsina N.P., Matveenko A.G., Barbitoff Y.A., Zhouravleva G.A., Bondarev S.A. (2020). Amyloid and amyloid-like aggregates: Diversity and the term crisis. Biochemistry.

[5] Alberti S., Halfmann R., King O., Kapila A., Lindquist S. (2009). A systematic survey identifies prions and illuminates sequence features of prionogenic proteins. Cell.

[6] Milles S., Huy Bui K., Koehler C., Eltsov M., Beck M., Lemke E.A. (2013). Facilitated aggregation of FG nucleoporins under molecular crowding conditions. EMBO Rep..

[7] Ader C., Frey S., Maas W., Schmidt H.B., Görlich D., Baldus M. (2010). Amyloid-like interactions within nucleoporin FG hydrogels. Proc. Natl. Acad. Sci. USA.

[8] Halfmann R., Wright J.J.R., Alberti S., Lindquist S., Rexach M. (2012). Prion formation by a yeast GLFG nucleoporin. Prion.

[9] Dultz E., Ellenberg J. (2010). Live imaging of single nuclear pores reveals unique assembly kinetics and mechanism in interphase. J. Cell Biol..

[10] Onischenko E., Weis K. (2011). Nuclear pore complex—A coat specifically tailored for the nuclear envelope. Curr. Opin. Cell Biol..

[11] Terry L.J., Wente S.R. (2009). Flexible gates: Dynamic topologies and functions for FG nucleoporins in nucleocytoplasmic transport. Eukaryot. Cell.

[12] Huerta-Cepas J., Szklarczyk D., Heller D., Hernández-Plaza A., Forslund S.K., Cook H., Mende D.R., Letunic I., Rattei T., Jensen L. (2019). eggNOG 5.0: A hierarchical, functionally and phylogenetically annotated orthology resource based on 5090 organisms and 2502 viruses. Nucleic Acids Res..

[13] Ahmed A.B., Znassi N., Château M.T., Kajava A.V. (2015). A structure-based approach to predict predisposition to amyloidosis. Alzheimers Dement..

[14] Dosztányi Z., Csizmók V., Tompa P., Simon I. (2005). The pairwise energy content estimated from amino acid composition discriminates between folded and intrinsically unstructured proteins. J. Mol. Biol..

[15] Dosztányi Z., Csizmok V., Tompa P., Simon I. (2005). IUPred: Web server for the prediction of intrinsically unstructured regions of proteins based on estimated energy content. Bioinformatics.

[16] Edgar R.C. (2004). MUSCLE: Multiple sequence alignment with high accuracy and high throughput. Nucleic Acids Res..

[17] Okonechnikov K., Golosova O., Fursov M., The UGENE team (2012). Unipro UGENE: A unified bioinformatics toolkit. Bioinformatics.

[18] R Core Team (2020). R: A Language and Environment for Statistical Computing.

[19] Pagès H., Aboyoun P., Gentleman R., DebRoy S. Biostrings: Efficient Manipulation of Biological Strings; R Package Version 2.56.0; 2020. https://bioconductor.org/packages/release/bioc/html/Biostrings.html.

[20] Wickham H., Averick M., Bryan J., Chang W., McGowan L., François R., Grolemund G., Hayes A., Henry L., Hester J. (2019). Welcome to the Tidyverse. J. Open Source Softw..

[21] Sivanathan V., Hochschild A. (2013). A bacterial export system for generating extracellular amyloid aggregates. Nat. Protoc..

[22] Sambrook J., Fritsch E., Maniatis T. (1989). Molecular Cloning: A Laboratory Manual.

[23] Hanahan D. (1983). Studies on transformation of *Escherichia coli* with plasmids. J. Mol. Biol..

[24] Kryndushkin D.S., Alexandrov I.M., Ter-Avanesyan M.D., Kushnirov V.V. (2003). Yeast [*PSI*^+^] prion aggregates are formed by small Sup35 polymers fragmented by Hsp104. J. Biol. Chem..

[25] Drozdova P.B., Barbitoff Y.A., Belousov M.V., Skitchenko R.K., Rogoza T.M., Leclercq J.Y., Kajava A.V., Matveenko A.G., Zhouravleva G.A., Bondarev S.A. (2020). Estimation of amyloid aggregate sizes with semi-denaturing detergent agarose gel electrophoresis and its limitations. Prion.

[26] Kushnirov V.V., Alexandrov I.M., Mitkevich O.V., Shkundina I.S., Ter-Avanesyan M.D. (2006). Purification and analysis of prion and amyloid aggregates. Methods.

[27] Studier F., Moffatt B.A. (1986). Use of bacteriophage T7 RNA polymerase to direct selective high-level expression of cloned genes. J. Biol. Chem..

[28] Serio T.R., Cashikar A.G., Moslehi J.J., Kowal A.S., Lindquist S.L. (1999). Yeast prion [*PSI*^+^] and its determinant, Sup35p. Methods Enzymol..

[29] Kajava A.V., Baxa U., Steven A.C. (2010). β Arcades: Recurring Motifs in Naturally Occurring and Disease-Related Amyloid Fibrils. FASEB J..

[30] Bondarev S.A., Zhouravleva G.A., Belousov M.V., Kajava A.V. (2015). Structure-based view on [*PSI*^+^] prion properties. Prion.

[31] Roche D.B., Villain E., Kajava A.V. (2017). Usage of a dataset of NMR resolved protein structures to test aggregation vs. solubility prediction algorithms. Protein Sci..

[32] Dewangan P.S., Sonawane P.J., Chouksey A.R., Chauhan R. (2017). The Nup62 coiled-coil motif provides plasticity for triple-helix bundle formation. Biochemistry.

[33] King C.Y., Tittmann P., Gross H., Gebert R., Aebi M., Wüthrich K. (1997). Prion-inducing domain 2–114 of yeast Sup35 protein transforms in vitro into amyloid-like filaments. Proc. Natl. Acad. Sci. USA.

[34] Eichner T., Radford S.E. (2011). Understanding the complex mechanisms of β2-microglobulin amyloid assembly. FEBS J..

[35] Chiti F., Dobson C.M. (2009). Amyloid formation by globular proteins under native conditions. Nat. Chem. Biol..

[36] Milles S., Mercadante D., Aramburu I.V., Jensen M.R., Banterle N., Koehler C., Tyagi S., Clarke J., Shammas S.L., Blackledge M. (2015). Plasticity of an Ultrafast Interaction between Nucleoporins and Nuclear Transport Receptors. Cell.

[37] Labokha A.A., Gradmann S., Frey S., Hülsmann B.B., Urlaub H., Baldus M., Görlich D. (2013). Systematic analysis of barrier-forming FG hydrogels from Xenopus nuclear pore complexes. EMBO J..

[38] Schmidt H.B., Görlich D. (2016). Transport selectivity of nuclear pores, phase separation, and membraneless organelles. Trends Biochem. Sci..

[39] Celetti G., Paci G., Caria J., VanDelinder V., Bachand G., Lemke E.A. (2020). The liquid state of FG-nucleoporins mimics permeability barrier properties of nuclear pore complexes. J. Cell Biol..

[40] Li C., Goryaynov A., Yang W. (2016). The selective permeability barrier in the nuclear pore complex. Nucleus.

[41] Fragasso A., de Vries H.W., Andersson J., van der Sluis E.O., van der Giessen E., Dahlin A., Onck P.R., Dekker C. (2021). A designer FG-Nup that reconstitutes the selective transport barrier of the nuclear pore complex. Nat. Commun..

[42] Onischenko E., Tang J.H., Andersen K.R., Knockenhauer K.E., Vallotton P., Derrer C.P., Kralt A., Mugler C.F., Chan L.Y., Schwartz T.U. (2017). Natively unfolded FG repeats stabilize the structure of the nuclear pore complex. Cell.

